# Different Shades of L1CAM in the Pathophysiology of Cancer Stem Cells

**DOI:** 10.3390/jcm9051502

**Published:** 2020-05-16

**Authors:** Marco Giordano, Ugo Cavallaro

**Affiliations:** Unit of Gynaecological Oncology Research, European Institute of Oncology IRCSS, 20128 Milan, Italy; marco.giordano@ieo.it

**Keywords:** L1CAM, cancer stem cells, chemoresistance, epithelial-mesenchymal transition, cancer therapy, cell adhesion molecule

## Abstract

L1 cell adhesion molecule (L1CAM) is aberrantly expressed in several tumor types where it is causally linked to malignancy and therapy resistance, acting also as a poor prognosis factor. Accordingly, several approaches have been developed to interfere with L1CAM function or to deliver cytotoxic agents to L1CAM-expressing tumors. Metastatic dissemination, tumor relapse and drug resistance can be fueled by a subpopulation of neoplastic cells endowed with peculiar biological properties that include self-renewal, efficient DNA repair, drug efflux machineries, quiescence, and immune evasion. These cells, known as cancer stem cells (CSC) or tumor-initiating cells, represent, therefore, an ideal target for tumor eradication. However, the molecular and functional traits of CSC have been unveiled only to a limited extent. In this context, it appears that L1CAM is expressed in the CSC compartment of certain tumors, where it plays a causal role in stemness itself and/or in biological processes intimately associated with CSC (e.g., epithelial-mesenchymal transition (EMT) and chemoresistance). This review summarizes the role of L1CAM in cancer focusing on its functional contribution to CSC pathophysiology. We also discuss the clinical usefulness of therapeutic strategies aimed at targeting L1CAM in the context of anti-CSC treatments.

## 1. Background

The L1 cell adhesion molecule (L1CAM, also known as CD171) was described for the first time by Schachner et al. in the central nervous system (CNS) [1]. In that context, L1CAM has been primarily implicated in the development and plasticity of the nervous system, where it plays a pivotal role in neuronal migration and differentiation, neurite outgrowth, axon guidance, fasciculation of axons and dendrites, myelination, and synaptogenesis [2,3,4]. Accordingly, the knockout of the murine gene results in profound neurological disorders [5,6], and mutations in human L1CAM are causally related to a spectrum of CNS defects that are collectively defined as L1 syndrome [7].

Following the discovery of L1CAM, other closely related cell adhesion molecules (CAMs) have been described, defining an L1 subfamily of which L1CAM is the archetype. In vertebrates, the L1 subfamily comprises four different members which share an analogous structural organization: Close Homolog of L1 (CHL1), Neuronal Cell Adhesion Molecule (NrCAM), Neurofascin and L1CAM itself [8]. All these proteins, in turn, belong to the Immunoglobulin superfamily of CAMs (Ig-CAMs), which owes its name to the presence of Ig-like domains in the extracellular portion of these proteins.

## 2. Molecular Characteristics of L1CAM

### 2.1. Structural Determinants of L1CAM Interactions

**Figure 1:**
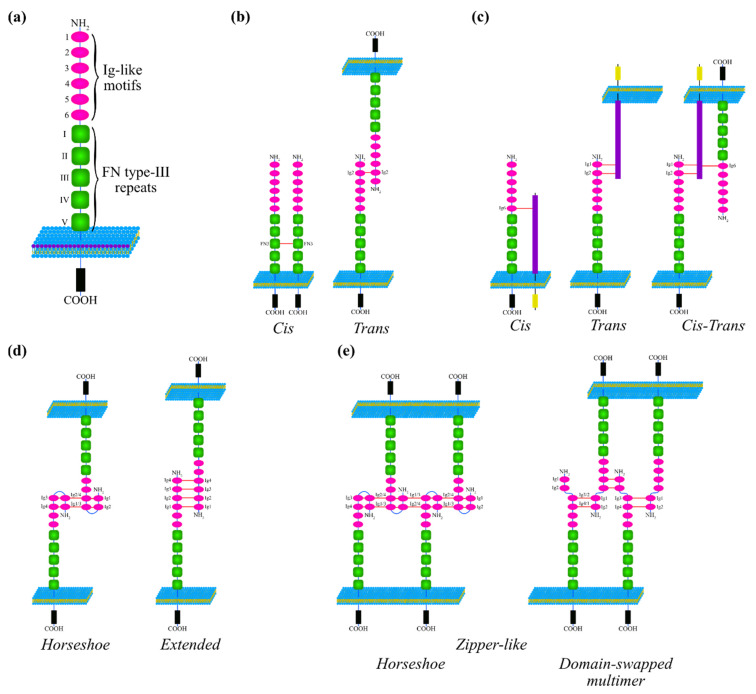
L1 cell adhesion molecule (L1CAM) structures and interactions. (**a**) Schematic structure of L1CAM, with the ectodomain comprising six Ig-like motifs (Ig1-Ig6, magenta ellipses) and five Fibronectin type-III repeats (FN1-FN5, green squares), the transmembrane (blue line) and the intracellular domain (black rectangle) (**b**) L1CAM homophilic interactions involve the FN3 repeat of two consecutive molecules (*cis*) that form hydrogen bonds (red line). When the molecules are exposed on two different cells, the involved residues can span between all the Ig-like motifs and FN2 or FN3 repeats (*trans*). For space limitations and clarity, only some homophilic bonds are displayed. See the main text for more details. (**c**) L1CAM heterophilic interactions involve almost all extracellular domains, with the specific domain depending on the partner. Only some examples of binding are shown here; more details are available in the main text. A generic partner is depicted as a transmembrane protein (violet rectangle: extracellular domain; yellow rectangle: intracellular domain). The two types of heterophilic interaction can be concomitant (*cis*-*trans*). (**d**) L1CAM can acquire different conformations during its interactions. The horseshoe structure is characterized by the bond between Ig-1 with Ig-4 and Ig-2 with Ig-3 that leads to a curvature of the molecule. Two horseshoe structures are connected by Ig-1 and Ig-2 repeats of adjacent molecules. The extended conformation involves bonds between the first four Ig structures of two neighbor L1CAM molecules. (**e**) More than two L1CAM molecules might be connected acquiring a very complex conformation (zipper-like). The domain-swapped multimer comprises interactions between Ig1 with Ig-4 and Ig2 with Ig-3. For space limitations and clarity, only interactions with full-length L1CAM are displayed. See text for more details.

L1CAM is a single-pass membrane glycoprotein with a molecular weight of 200–220 kDa which exhibits three different portions: an extracellular domain, a transmembrane domain and a highly conserved cytoplasmic domain (Figure 1a) [9].

The ectodomain comprises six N-terminal Ig-like motifs (Ig1–Ig6) followed by five fibronectin type III repeats (FN1–FN5) [4,8]. These structural elements and their dynamic arrangements are crucial to establish and drive the multiple interactions of L1CAM. Indeed, L1CAM exerts its function through inter-molecular interactions that can be either homophilic (i.e., L1CAM-L1CAM) or heterophilic with different partners. L1CAM can engage in *cis*-interactions, binding to another protein at the surface of the same cell (Figure 1b,c), *trans*-interactions with a protein localized on an adjacent cell (Figure 1b,c), or *cis*/*trans*-interactions where both events can occur simultaneously (Figure 1c) [10]. When these interactions take place, the proteins involved may assume different structural conformations in order to facilitate their binding that are illustrated in Figure 1. Su et al. predicted that L1CAM can acquire a horseshoe quaternary structure (Figure 1d). Another possible conformation that L1CAM acquires is the so-called extended quaternary structure (Figure 1d). Interestingly, these four domains are critical for neurite outgrowth and L1CAM adhesive properties, even though additional sequences within all the six Ig domains are required for an optimal activity [11,12,13]. Cryo-electron tomography studies also revealed a more complex mode of homophilic interaction whereby horseshoes from L1CAM proteins on opposing membranes meet as *trans* pairs, forming a lattice that is stabilized by protein-carbohydrate and carbohydrate-carbohydrate interactions (Figure 1e) [14]. The existence of such horseshoe-dependent structures is still controversial as some researchers attribute this conformation to a complicated mixture of other quaternary structures at a higher level of complexity rather than to a structure *per se* [14].

### 2.2. L1CAM Interactions

L1CAM is devoid of enzymatic activity and, therefore, needs molecular effectors for transducing intracellular signals and regulating the multiple processes in which it is involved. In this context, L1CAM often couples with other cell-surface molecules that, instead, have the capability to activate a downstream signaling. The proteins involved in a functional and/or physical interaction with L1CAM belong to different classes including other Ig-CAMs (such as NCAM), proteoglycans (e.g., neurocan), integrins, extra cellular matrix proteins (laminin), co-receptors (neuropilin-1), cytoskeletal proteins (ankyrin), and Receptor Tyrosine Kinases (RTKs) such as Fibroblast Growth Factor (FGF) and Epidermal Growth Factor (EGF) receptors. The main features of these interactions are summarized in Table 1.

#### 2.2.1. L1CAM Interacting Partners and Functional Implications

L1CAM and NCAM interact with each other in *cis* [18,19,29]. This interaction allows L1CAM to bind other L1CAM molecules in *trans* and, therefore, has been termed “assisted homophilic binding”. This binding has synergistic effects on L1CAM-mediated cell aggregation and adhesion in neuroblastoma cells [19,29]. L1CAM can also interact with neurocan [20,30]. The Ig6 motif of L1CAM contains the highly conserved aminoacidic sequence Arg-Gly-Asp (RGD) that is crucial for the *cis* interaction of L1CAM with α_v_β_3_, α_v_β_1_, α_5_β_1_, α_v_β_5_, α_IIB_β_3_ integrins [21,22,31]. The FN3 repeat is also involved in the binding of L1CAM with integrins, in particular with α_5_β_1_, α_v_β_3_, α_9_β_1_ [17]. The interaction with laminin occurs, although not exclusively, via the human natural killer-1 (HNK-1) carbohydrate [23]. The binding between L1CAM and Neuropilin-1 (NRP1 or NP-1) requires the small aminoacidic motif FASNKL [10,24]. Castellani and collaborators showed that the switching of semaphorin-3A (Sema3A)-induced axonal repulsion into attraction depends on *cis* vs. *trans* interactions of L1CAM with NP-1, respectively. In this scenario, L1CAM and NP-1 could be considered as co-receptors for Sema3A. Hence, this *cis* interaction is required as part of the Sema3A receptor complex and is necessary for the switching mechanism [10]. The cytoskeletal protein ankyrin is a prominent intracellular partner of L1CAM, and their interaction occurs through the highly conserved amino acid motifs LADY and FIGQY [25,26]. In neurons, L1CAM interaction with ankyrin is critical for the synaptic targeting of retinal axons and it also induces, in co-operation with EphrinB signaling, axon branch attraction in vivo [32]. The first RTK proposed to interact with L1CAM (and other adhesion molecules) is the fibroblast growth factor receptor (FGFR) [33]. Among the four members of the FGFR family, the direct interaction with L1CAM so far has been demonstrated only for FGFR1 [27,34]. L1CAM can also bind all the members of the EGFR family (erbB1-erbB4) [35]. When L1CAM *trans* interacts with EGFR, the binding is very weak and is not sufficient *per se* for EGFR autophosphorylation, even though a tyrosine kinase activity was detected at cell contact sites in *D. melanogaster* [36]. This implies that *cis* interactions between the two types of molecules may be required to enhance L1CAM-induced activation of EGFR. To note, a *cis* contact with erbB3 has been described in vivo [35].

#### 2.2.2. The Regulation of L1CAM Interactions via Phosphorylation of the Cytoplasmic Tail

A key feature of L1CAM that can modulate its interactions is the phosphorylation of its cytoplasmic domain. Three kinases have been implicated in this process: ERK2, c-SRC and PKCα. The first to be identified was ERK2, a key player in the mitogen-activated protein kinase (MAPK) signaling, that interacts physically with L1CAM. Schaefer et al. have found that ERK2 is able to phosphorylate L1CAM at Ser^1204^ and Ser^1248^ n vitro, and suggested that this phosphorylation regulates the binding of L1CAM to ankyrin [37]. L1CAM and ERK2 crosstalk is bidirectional, since L1CAM stimulates ERK2 activation and MAPK signaling, most likely via FGFR. L1CAM-mediated ERK2 activation has been proposed to occur upon L1CAM endocytosis since both L1CAM and ERK2 are present in endocytic vesicles [37]. The second kinase implicated in L1CAM phosphorylation is the nonreceptor tyrosine kinase c-SRC. The latter phosphorylates the Tyr^1176^ residue within the YRSLE motif [38] that is required for endocytosis of L1CAM via clathrin-coated pits [39]. The authors have shown that Tyr^1176^ phosphorylation prevents L1CAM binding to clathrin-associated AP-2 complex, suggesting a model whereby transient dephosphorylation of the YRSLE motif allows L1CAM endocytosis. Finally, protein kinase C-alpha (PKCα) phosphorylates the Thr^1172^ residue within the L1CAM cytoplasmic domain, thus regulating important properties of pancreatic adenocarcinoma cells such as motility [40,41]. Notably, Thr^1172^ phosphorylation promotes conformational changes that, in turn, influence the interactions of L1CAM even within its extracellular portion. For example, the binding to integrins is profoundly affected by the phosphorylation status of Thr^1172^ [40,41]. It is worth mentioning, however, that these studies relied principally on the use of recombinant proteins, and therefore need further validation in more physiological settings.

In summary, L1CAM engages in interactions with a myriad of molecules that impinge on several signal transduction pathways which, in turn, orchestrate fundamental aspects of cell physiology.

### 2.3. Proteolytic Processing of L1CAM

**Figure 2:**
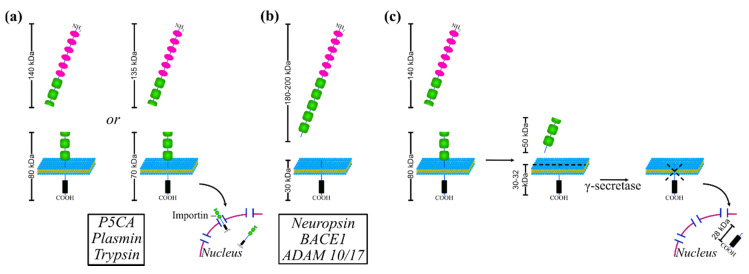
Processing of L1CAM. (**a**) Full-length L1CAM undergoes a cleavage inside the FN3 repeat by serine proteases PC5A, plasmin or trypsin. Thus, a soluble fragment of about 135-140 kDa (top) and another of 75-80 kDa are produced. The former is released while the remaining fragment, which comprises part of the ectodomain, the transmembrane domain and the cytoplasmic domain, is internalized and imported into the nucleus via importin. (**b**) L1CAM is cleaved closer to the membrane by neuropsin, BACE1 or ADAM10/17 proteases generating a soluble fragment of about 180-200 kDa (top) and one of 30 kDa. (**c**) The processes described in (**a**,**b**) can occur sequentially. First, L1CAM is cleaved inside the FN3 repeat; a second cut occurs close to the membrane producing a fragment of 50 kDa and another of 30-32 kDa, which remains anchored to the membrane. The latter is further processed by γ-secretase and is released from the plasma membrane, eventually translocating into the nucleus.

By analogy to other Ig-CAMs, L1CAM can be cleaved by several proteinases, a process that regulates both the cell-autonomous and non-cell-autonomous signaling of the protein [42]. These proteolytic events and the generated L1CAM fragments are summarized in Figure 2.

A cleavage site in the FN3 domain of L1CAM is recognized by the serine proteases PC5A, plasmin and trypsin [43,44,45] (Figure 2a). The proteolytic action of these enzymes generates two fragments, one of about 140-kDa and one of 80-kDa. Lutz and collaborators demonstrated the production of alternative fragments of 135-kDa (soluble) and 70-kDa that comprise the intracellular and transmembrane domains and part of the extracellular domain (Figure 2a). The nuclear translocation of the 70-kDa fragment, which involves sumoylation of the residue Lys^1172^, is mediated by importin, results from trafficking via endosomes and depends on the presence of a nuclear localization signal encompassing Lys^1147^. However, the authors did not investigate the possible role of nuclear L1CAM fragment in gene transcription [46]. Besides the serine protease-mediated processing, the ectodomain of L1CAM can be cleaved closer to the membrane, thus producing a bigger fragment of about 180-200 kDa (Figure 2b). The proteases responsible for such process are Neuropsin, β-secretase 1 (BACE1) and the metalloproteases ADAM10 and ADAM17 [47,48,49,50,51]. Notably, the two types of processing may occur sequentially (Figure 2c). First, a fragment of 140-kDa is produced upon cleaving inside the FN3 domain. The remaining portion of the ectodomain is further processed to produce a soluble fragment of 50-kDa [43]. Whatever protease accounts for the iuxtamembrane cleavage of L1CAM, in addition to the above mentioned 180-200 kDa portion of the ectodomain, a 30-32 kDa fragment is always produced. This, in turn, is further processed by γ-secretase resulting in a smaller fragment of 28 kDa that is released from the membrane, translocates into the nucleus and triggers gene transcription [51]. However, soluble L1CAM can also derive from events other than proteolytic cleavage. Indeed, Angiolini et al. have recently described a novel, endothelial-specific isoform of L1CAM, that results from a peculiar alternative splicing of its pre-mRNA. In particular, the splicing factor NOVA2 induces the skipping of the exon that encodes the transmembrane domain, generating a soluble L1CAM that retains both the extracellular and the cytoplasmic domains and is endowed with angiogenic activity [52].

## 3. Clinical Relevance of L1CAM in Cancer Diagnosis and Prognosis

The definition of L1CAM’s role in cell motility and plasticity within the nervous system prompted many groups to investigate whether the protein exerts an analogous role in different contexts. Numerous studies focused on the function of L1CAM in tumor-related processes. Indeed, L1CAM has emerged as a causal factor in tumor invasion and metastasis. Such a role is extensively described in a number of excellent reviews [42,53,54] and will not be discussed here. We will mainly focus on the clinical relevance of L1CAM in cancer patients, and in particular on the correlation of its levels with the prognosis, the diagnosis and other clinical parameters in certain cancer types (Table 2).

The potential of L1CAM as a prognostic factor has received great attention from investigators working on gynecological tumors. Among these, the tumor in which the prognostic role of L1CAM has been investigated most extensively is endometrial cancer.

Bosse et al. [55] evaluated L1CAM levels in a large retrospective cohort of early stage endometrial cancer. They have found that patients with >10% tumor cells positive for L1CAM had a remarkably shorter overall survival than those below that threshold. Smogeli et al. [56] found that, in the subgroup of patients who did not receive adjuvant chemotherapy, L1CAM expression was significantly associated with shorter disease-free survival and with risk of relapse in univariate analysis. In a different study that analysed biopsies from 1134 endometrial cancer patients, L1CAM high expression predicted poor disease-specific survival – defined as time from surgery to death – both in the entire cohort and among low-risk patients, who normally receive no or limited adjuvant treatment [57]. Moreover, high expression of L1CAM correlated significantly with the occurrence of lymph node metastases, both in the whole patient population and in the low-risk subgroup. In addition, the authors evaluated the serum level of soluble L1CAM (sL1CAM) in preoperative samples from a subgroup of 372 patients with endometrial cancer. A higher amount of sL1CAM was detected in cancer patients with respect to healthy controls, in line with previous studies [61]. High levels of sL1CAM predicted poor disease-specific survival in both the entire cohort and in the low-risk group. sL1CAM levels were also predictive for lymph node metastasis in the entire cohort. However, once adjusted for age, FIGO stage, histologic type and grade, circulating sL1CAM levels failed to exhibit independent prognostic power.

L1CAM has also been assessed as a biomarker for preoperative risk factor stratification of endometrial carcinoma. However, it did not significantly improve risk stratification when compared to classical factors [72], implying that L1CAM should not be used to plan preoperative treatment. Another study showed that L1CAM is highly expressed in recurrent respect to non-recurrent endometrial cancer and correlates with lower disease-free survival [58].

A multicentric study named ENITEC, which involved 10 different European centers, assessed L1CAM expression in 1199 cases that included early and late-stage endometriod endometrial cancers (EECs) as well as non-endometriod endometrial cancers (NEECs) [59]. In early-stage EECs, L1CAM expression was associated with grade 3 histology and lymphovascular space invasion (LVSI) while in late-stage EECs its expression correlated also with the presence of nodal disease. Moreover, L1CAM levels were associated with shorter disease-free and overall survival in both early and late-stage EECs. Very recently, L1CAM expression was also found to correlate with distant recurrence in early-stage endometrial cancer patients with negative peritoneal cytology, a subgroup that normally has a relatively good prognosis [60].

**Figure 3:**
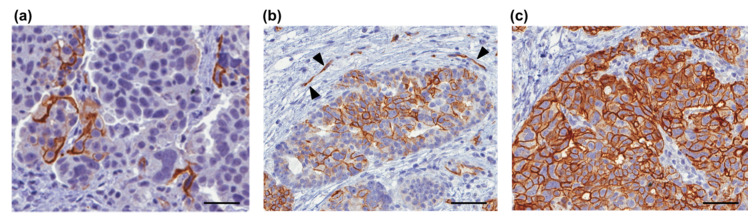
L1CAM levels in ovarian cancer. Three examples of high-grade serous ovarian cancer with different levels of L1CAM are shown. L1CAM is visualized by immunohistochemistry on formalin-fixed paraffin-embedded specimens. The protein is localized on the cancer cell membrane with levels that range from (**a**) a minority of cells to (**c**) most of the tumor mass. The arrowheads in (**b**) show ovarian cancer-associated vessels that are L1CAM-positive as previously described [56]. Scale bars: (**a**), 25 µm; (**b**,**c**) 50 µm.

In the context of gynecological oncology, the clinical utility of L1CAM has also been extensively studied in ovarian cancer (OC). In this tumor type, L1CAM shows a wide range of expression, from a small fraction of cancer cells to high levels in most of the tumor mass (Figure 3a–c).

Fogel et al. reported the expression of L1CAM in poorly differentiated OC, where it acts as a biomarker of worse prognosis [61]. Other groups have then confirmed the association of L1CAM with bad outcome [42,62], although some studies have questioned the prognostic value of L1CAM in OC [73]. Of note, L1CAM was consistently detected at the invasive front of OC [61,62,64], supporting its role in tumor invasion.

In type-I ovarian cancer, often confined to the ovary and resulting in good prognosis, L1CAM was assayed in patients with either endometriod or clear cell histotype [63]. The authors showed that L1CAM levels correlated with poor disease-specific overall survival and disease-free survival in endometrioid, but not in clear cell ovarian carcinomas. Moreover, overall survival was worse in early-stage patients with high L1CAM levels, making L1CAM a potential stratification marker for a high-risk subgroup among these putative good-prognosis patients. Although L1CAM alone did not result in multivariate analysis as an independent prognostic factor for overall disease survival, its positivity was associated with incomplete response to primary therapy in endometroid ovarian cancer but, once again, not in the clear cell histotype [63]. Finaly, Altevogt and collaborators measured the levels of both membrane and soluble L1CAM in a cohort of high grade serous ovarian cancer patients [64]. They found that soluble L1CAM isolated from patients’ ascitic fluid correlated with poor outcome in terms of PFS and showed a trend toward prolonged OS.

The correlation of L1CAM expression with poor prognosis is by no means limited to the gynecological cancers described above. Indeed, L1CAM has been defined as a negative prognostic factor in melanoma [65], breast cancer [66], gastric cancer [67], colon cancer [68], pancreatic cancer [69], non-small cell lung cancer [70], kidney cancer [71], etc., as elegantly reviewed elsewhere [42,53,54].

In summary, the clinical utility of L1CAM as a diagnostic and prognostic factor has emerged from many studies in different types of cancers, albeit with some controversial observations, which is in line with its involvement in malignancy-associated features of tumor cells [42,53,54].

## 4. L1CAM Mechanism of Action in Stemness and in Stem-Related Processes

A common fate of all cancer patients, especially for those who have been diagnosed at late stage, is that they undergo chemotherapy and/or radiotherapy. However, even in case of a satisfactory response of the primary tumor to the treatments, many patients experience a disease relapse, and the scenario is even worsened by the acquisition of chemoresistance which makes the recurrent tumor refractory to standard therapies. Tumor relapse and drug resistance are commonly attributed to a subset of cells that, taking advantage of an armory of biological weapons, evade chemotherapy with diverse resistance mechanisms. Such cell subpopulation appears related to cancer stem cells (CSC) [74]. This hypothesis is supported by certain characteristics of CSC such as: a low proliferative potential that makes them insensitive to chemotherapy which usually targets actively dividing cells; increased levels of molecular pumps that efflux drugs out of the cell; adaptation to inflammation; efficient DNA repair; and altered apoptotic mechanisms [75].

CSC have been the subject of intense debate and controversy over the last two decades. Some researchers refer to them as a transient state of tumor cells rather than a discrete subpopulation [76], a concept strictly related to tumor cell plasticity; others are even questioning their existence [77,78]. According to many investigators, much of the controversy and skepticism around CSC derives from a semantic issue, namely what criteria must be considered to define bona fide CSC [79]. As a matter of fact, a subset of cancer cells with distinct tumor-initiating ability does evade the conventional therapies and fuels tumor recurrence [80,81]. These features of CSC account for the common use of the term cancer-initiating cells (CIC) as a synonym [82]. Collectively, there is a body of experimental and clinical evidence that supports the existence and the pro-malignant function of cancer cells with stem-like features [79].

### 4.1. L1CAM Function in CSC and Its Contribution to Cancer Stemness-Associated Processes

L1CAM has been linked to many CSC-related processes, yet only a few studies have demonstrated its direct involvement in stemness. Tumor recurrence and metastasis is accompanied and fueled by specific biological processes, such as the acquisition of chemoresistance and EMT, that have been found to be intimately associated with cancer stemness [83]. Hence, it is not surprising that, due to its causal role in CSC biology, L1CAM has been also implicated in these processes in cancer, even though these studies were not always conducted on CSC subpopulations.

#### 4.1.1. Glioblastoma

Similarly to what occurred during its discovery, the first connection of L1CAM with CSC was found in the central nervous system [84]. Through a loss-of-function approach, the authors showed that silencing L1CAM in glioblastoma stem cells (GSC), defined by CD133 expression, reduced their growth and survival both in vitro and in vivo. They also described the underlying mechanism that entails the loss of Olig2, caused by L1CAM silencing, and the upregulation of cyclin-dependent kinase inhibitor p21^WAF1/CIP1^, which acts as a tumor suppressor [84]. In another study, Cheng and coworkers showed that L1CAM regulates DNA damage responses and radiosensitivity of CD133^+^ or CD15^+^ GSC through the nuclear translocation of its intracellular domain [85]. In particular, L1CAM expression is induced by DNA damage, and is critical for the activation of ataxia telangiectasia mutated (ATM) and of the downstream checkpoint proteins Chk2, Rad17, and Chk1, which repair the DNA as part of the DNA damage response signaling pathway [86]. Mechanistically, the portion of L1CAM involved in this process is the highly conserved cytoplasmic domain that acts as signal transducer upon DNA damage, regulating NBS1 expression through c-Myc to enhance DNA damage checkpoint activation. The findings discussed above might implicate L1CAM as a potential marker of glioblastoma CSC. Indeed, L1CAM has been frequently employed to identify stem-like population in glioblastoma [84,85,87,88]. Nevertheless, CSC-related functions, such as tumor initiation and self-renewal abilities, have not been defined yet by comparing L1CAM-positive and L1CAM-negative subpopulations of glioma cells [89]. Thus, the role of L1CAM as a GSC marker remains to be conclusively demonstrated.

Glioblastoma provides also a prototypical example of L1CAM’s role in chemoresistance [90]. Held-Feindt et al. started from the earlier observation that TGF-β1 is a potent inducer of L1CAM expression in tumor cells [91,92]. Notably, these authors employed clinically relevant models of primary tumor cells and cultivated them as suspension neurospheres as a tool to enrich for glioblastoma stem-like cells. They showed that L1CAM promoted chemoresistance to temozolomide, which was mediated by TGF-β1 and led to the down-regulation of caspase-8 in both stem-like and bulk glioblastoma cells. These data raised the question of whether chemoresistance is restricted to CSC compartment only or rather it can also be ascribed to non-stem subpopulations. However, it is appropriate to point out that the cells used in the study derived from the differentiation of stem-like cells. It is conceivable, in this case, that such differentiated cells might have retained chemoresistance as an imprinting from their stem state.

#### 4.1.2. Colorectal Cancer

The identification of L1CAM as a target of β-catenin/TCF signaling in colorectal cancer (CRC) [93,94] paved the way to the definition of its role in CRC stem cells. Gavert et al. [95] observed initially that, while the ectopic expression of L1CAM enhanced CRC metastasis, likely via the activation of NF-κB signaling, L1CAM was not co-expressed with the CRC stem cell markers EpCAM, CD133 and CD44. However, L1CAM was subsequently reported to be expressed in LGR5^+^ CRC stem cells, where it promoted metastatic dissemination through the induction of the clusterin gene (*CLU*), an event independent from NF-κB signaling [96]. In particular, L1CAM enhanced the transactivation of *CLU* by the transcription factor STAT-1. The link between L1CAM and STAT-1, however, remains to be defined at the molecular level. The role of L1CAM in CRC stemness has also been investigated by Basu et al. who focused on the Wnt target gene and transcription factor Achete scute-like 2 (ASCL2), a key regulator of stemness that is exclusively expressed in LGR5^+^ intestinal stem cells [97,98]. Basu et al. implicated ASCL2 as an L1CAM effector in CRC progression [99]. Indeed, L1CAM expression in human CRC cells dramatically increased the expression of ASCL2 which, in turn, was required for L1CAM-induced CRC cell proliferation, motility and tumorigenesis. Finally, L1CAM and ASCL2 were found to co-localize in human CRC tissue, suggesting a possible cooperation in conferring a more invasive phenotype to CRC cells. A very recent study has provided compelling evidence that high L1CAM expression marks a subpopulation of CRC cells with tumor propagation, metastasis-initiating and chemoresistance features [100]. Intriguingly, L1CAM^high^ cells partially overlapped with LGR5^+^ CRC stem-like cells. In this context, L1CAM expression was not only a biomarker but also a prerequisite for metastasis initiation and chemoresistance. Ganesh et al. also provided mechanistic insights into the upregulation of L1CAM by showing that the loss of E-cadherin-dependent epithelial integrity releases L1CAM from the transcriptional repression operated by REST, a factor that prevents L1CAM expression in non-neuronal tissues [100]. Very recently Fang et al. correlated L1CAM with pERK 1/2 levels in CRC lymph node metastasis [101]. The authors showed that L1CAM expression in tissue samples increased from poorly differentiated through well differentiated CRC reaching the highest levels in metastatic CRC tissue. The same behavior was observed for pERK 1/2. Future work should clarify whether the positive correlation between L1CAM and pERK levels reflects a functional link and whether L1CAM-regulated ERK signaling contributes to CRC dissemination.

#### 4.1.3. Pancreatic Cancer

L1CAM-associated chemoresistance has also been proposed in pancreatic cancer. Lund and collaborators generated a pancreatic carcinoma cell line resistant to the chemotherapeutic 5-Fluorouracil (5-FU) and, upon transcriptomic profiling, identified L1CAM interaction pathway as one of the top-ranking hits among 319 upregulated genes [102]. Silencing L1CAM resulted in decreased invasiveness of the 5-FU resistant cell line. Driven by their microarray data and by previous observation in pancreatic cancer [92], the authors knocked down Slug and β-catenin in chemoresistant cells and found that the former, but not the latter, modulates L1CAM protein levels. Yet whether a Slug/L1CAM axis accounts for chemoresistance in pancreatic carcinoma remains to be investigated.

#### 4.1.4. Gynecological Cancers

Among tumors of the gynecological tract, the role of L1CAM in cancer stemness has been studied in ovarian and endometrial carcinoma. A study conducted on ovarian cancer cell lines revealed that L1CAM, in combination with CD133, marks a subpopulation of ovarian CSCs [103]. In light of the known correlation of L1CAM with ovarian cancer aggressiveness [61,62], these findings, once confirmed in patient-derived tissue, might implicate L1CAM/CD133-positive CSC in the malignant properties of this tumor type. Furthermore, the repertoire of ovarian CSC-associated markers is still a highly debated and controversial issue [82], and L1CAM may offer a new tool for the unequivocal identification of this elusive cell population. The study on L1CAM+/CD133+ ovarian CSCs showed also, through the genetic manipulation of ovarian cancer cell lines, that L1CAM is causally involved in CSC-associated radioresistance as well as in self-renewal and tumor initiation. Further research, however, should aim at elucidating the underlying molecular mechanisms.

In ovarian cancer cells, the L1CAM gene was found to be under the regulation of TWIST1, a transcription factor that is causally linked to increased tumorigenicity as well as resistance to cisplatin [104]. Indeed, L1CAM was upregulated upon the forced expression of TWIST1 and, more important, it was required for TWIST1-induced chemoresistance. Mechanistically, upon cisplatin treatment, L1CAM silencing partially prevented Akt activation, which was a key player in cisplatin resistance of ovarian cancer cells. These data, therefore, pointed to a TWIST1/L1CAM/Akt signaling pathway that drives chemoresistance in ovarian cancer.

As discussed earlier, L1CAM correlates with malignancy in endometrial cancer. In agreement with those observations, recent studies have established a link between L1CAM and endometrial cancer stemness [105]. The authors observed first that L1CAM promotes epithelial-mesenchymal transition (EMT) as exemplified by the concomitant downregulation of E-cadherin and induction of vimentin. Given the well-established association between EMT and cancer stemness [83], the authors then investigated CSC-related features. They found that L1CAM-expressing cells exhibit resistance to *anoikis* and higher clonogenic potential in non-adherent conditions, two peculiar features of CSC [82]. Moreover, L1CAM expression was accompanied by the upregulation of Musashi-1 and CD133, both considered to be CSC markers in endometrial cancer [106].

#### 4.1.5. Retinoblastoma

Recently, L1CAM has been reported to be both sufficient and necessary for conferring chemoresistance to retinoblastoma, the most common intraocular cancer in children [107]. Proteins related to apoptosis and multi-drug resistance (MDR) are frequently involved in the resistance of cancer cells and CSCs to chemotherapy [108,109,110]. Along this line, L1CAM depletion in retinoblastoma cells resulted in a marked increase of the pro-apoptotic proteins which cleaved caspase-3 and cytochrome C, whereas the anti-apoptotic proteins Bcl-2, Bcl-xL, and pro-caspase-3, were reduced. Moreover, the drug efflux pumps ABCA1, ABCB1, ABCC2, and ABCG2 were significantly reduced in L1CAM-depleted cells whereas L1CAM overexpression increased their levels.

#### 4.1.6. L1CAM Impact on Stemness-Related Features of Tumor Microenvironment

The contribution of L1CAM to tumor development can also occur via the modulation of stemness-related properties within the tumor microenvironment, which in turn modulates cancer cell behavior in a non-autonomous manner. For example, in tumor endothelium L1CAM promotes endothelial-to-mesenchymal transition (EndMT), a phenomenon reminiscent of EMT [111]. In this context, L1CAM induces the expression, among many other genes, of KLF4 and CD44, both well-known as stemness-associated factors. EndMT generates cells that retain the properties of multipotent stem cells and can differentiate into several cell types (e.g., fibroblast, pericyte, bone, etc.) [112]. Furthermore, EndMT produces key cellular components of the tumor microenvironment such as cancer-associated fibroblasts that support tumor progression [113]. This might open a novel scenario whereby L1CAM could also orchestrate stemness in the tumor microenvironment, adding a further layer of complexity to its role in cancer progression.

#### 4.1.7. L1CAM Expression Obtained by Omics Data Unveiled its Involvement in CSC Processes

By comparing cancer stem cell genetic profile with their non neoplastic counterpart, L1CAM emerged very frequently altered. These data endorse the crucial role of the adhesion molecule in cancer stem cells.

Nakata et al. demonstrated that LGR5 gene is associated with stem features in glioblastoma [114]. A transcriptomic analysis of LGR5-silenced glioblastoma stem cells showed that L1CAM is downregulated and therefore is regulated by LGR5, altough the molecular mechanism remains to be elucidated. In another study, Okawa et al. profiled and compared the proteome and secretome of glioblastoma multiforme stem cells (GNS) and normal neural stem cells [115]. The authors found that several CSC markers were enriched in the glioblastoma stem cell population, and L1CAM was one of them. Interestingly, L1CAM was found aberrantly expressed both in the secretome and in the cell-associated proteome of GNS, suggesting that its function is specifically involved in the stem cell compartment of glioblastoma.

Finally, Gemei et al. analyzed the genetic profile of the 3AB-OS osteosarcoma cell line that is an immortalized CSC line and represents the stem component of MG63 parental cell line used for genetic comparison [116]. L1CAM was among the genes whose expression increased in the c3AB-OS cell line with respect to the differentiated MG63.

A transcriptomics profile combined with high-throughput flow cytometry was conducted on self-renewing (i.e., stem-like) and non-self-renewing cells from the sonic hedgehog (SHH) subgroup of medulloblastoma. In this case, unlike the studies reported above, L1CAM was found specifically downregulated in CSCs at both gene and protein levels [117]. This suggests that the expression of L1CAM as a CSC-associated marker is tumor type-dependent, and in certain tumors L1CAM can even mark selectively the non-stem cancer cell population.

In conclusion, several studies have provided compelling evidence that implicates L1CAM, directly or indirectly, in the regulation of CSC pathophysiology. Yet none of them have conclusively indicated or demonstrated L1CAM to be a CSC (or CIC) marker. Therefore, the jury is still out on the use of L1CAM for the isolation, identification and characterization of the CSC compartment in clinically relevant settings.

### 4.2. Beyond Cancer: L1CAM in Normal Stem Cells

Besides the role of L1CAM in cancer stemness, it is worth mentioning that a few studies have implicated the molecule also in normal stem cells. Son and collaborators [118] demonstrated that the stem cell markers octamer-binding transcription factor 4 (Oct4), Nanog, sex-determining region Y–box 2 (Sox2), forkhead box protein D3 (FoxD3), and SSEA-3 were downregulated in L1CAM-depleted human embryonic stem cells (hESC). Conversely, the same genes resulted in being upregulated in L1CAM-overexpressing hESC. L1CAM-depleted hESC displayed also an increased expression of lineage markers (i.e., Olig2, GFAP, Pax6 as ectoderm marker; CD31, T-Branch, LEF1 as mesoderm marker; FOXA2, GATA4, SOX17 as endoderm marker). Moreover, the authors showed that L1CAM is essential for hESC pluripotency. In particular, when normal H9 cells were differentiated into embryoid bodies (EBs) most lineage markers did not increase in L1CAM-depleted cells whereas they were upregulated in control cells. Mechanistically, FGFR1 appeared to be involved in this process, although the exact mechanism remains to be elucidated. L1CAM formed a complex with FGFR1 and a reduced activation of FGFR1, ERK and AKT was observed in L1CAM-depleted cells [118]. Another study, performed on murine ESC (mESC), showed that L1CAM is required for neuronal differentiation of mESC and depends on the fucosyltransferase FUT9 and sialyltransferase ST3Gal4 through a signaling pathway that involves the activation of phospholipase C-gamma (PLCγ) [119]. It remains unclear whether the discrepancy between the impact of L1 in hESC, where it sustains stemness, and mESC, where it promotes differentiation, reflects a species-specific role of L1 in ESC or rather depends on different experimental conditions between the two studies.

Further complexity is added by a recent study on L1CAM in human neural progenitors (hNP). In this context, the ectopic expression of L1CAM extracellular domain altered differentiation and motility in hNP without affecting cell proliferation [120]. L1CAM-expressing hNP lost their progenitor status and became committed to differentiation. Interestingly, these authors co-cultured hNP with chick embryo brain cells to assess phenotype alterations in a more complex microenvironment. In these culture conditions L1CAM ectodomain again promoted the loss of the progenitor phenotype and induced the differentiation of hNP towards astrocytes. Taken together with the stemness-sustaining role of L1CAM in hESC (see above), these findings on promoting commitment in hNP might imply that the outcome of L1CAM expression is cell context-dependent.

## 5. L1CAM as a Therapeutic Target

**Figure 4:**
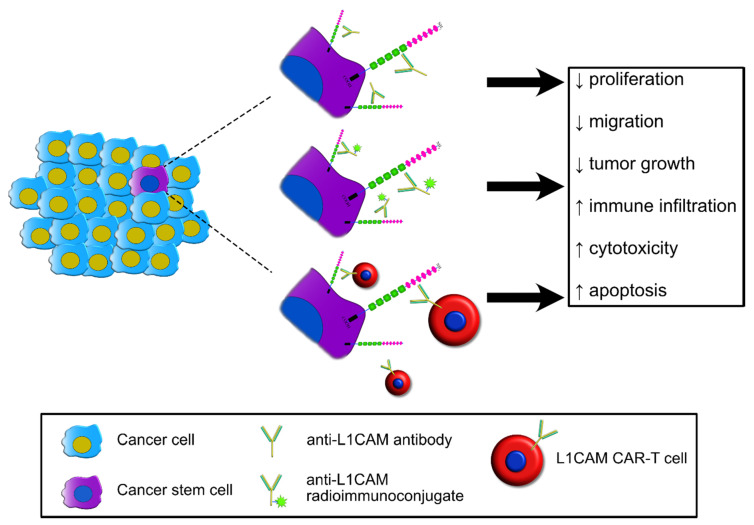
L1CAM is a viable therapeutic target in cancer. Three strategies for targeting L1CAM in cancer cells are depicted. Such strategies are based on neutralizing antibodies, radioimmunoconjugates or chimeric antigen receptor-redirected T (CAR-T) cells. These treatments, alone, or in combination with chemotherapy, result in the reduction of tumorigenicity due to increased apoptosis and cytotoxic effects, possibly accompanied by increased immune infiltration into the tumor site. Based on the expression and function of L1CAM in cancer stem cells, these L1CAM-targeted approaches may prove effective anti-CSC strategies, as illustrated in the figure. See the main text for more details.

The expression pattern of L1CAM in cancer and its functional role in CSC point to this molecule as a viable target for novel therapeutic strategies. While no attempts have been reported yet to test the druggability of L1CAM in CSC, several studies have supported the potential of L1CAM-targeted treatments in different tumor types, as summarized in Table 3. The three main strategies that have been designed for this purpose are illustrated in Figure 4 and described below.

### 5.1. Monoclonal Antibodies

In many cases, L1CAM-targeted approaches have relied on neutralizing antibodies (Figure 4). The latter, for example, exhibited a remarkable potential as antitumor agents in preclinical models of ovarian cancer. Arlt et al. induced antibody-mediated reduction of proliferation and migration in vitro as well as tumor growth in vivo using two independent anti-L1CAM monoclonal antibodies (chCE7 and L1-11A) [121]. We have also reported a reduction of proliferation in IGROV1 cells upon anti-L1CAM monoclonal antibody CE7 treatment [62]. Wolterink and coworkers generated novel monoclonal L1CAM antibodies [122]. The selected clone L1-9.3 inhibited SKOV3ip tumor growth, increasing mouse survival. The transcriptome analysis of treated mice revealed also that L1-9.3 treatment could interfere with apoptotic and tumor growth pathways. To note, the treatment with L1-9.3 was accompanied by massive monocyte infiltration, and monocyte depletion via clodronate liposomes abolished the therapeutic effect of the antibody. This role of monocytes in the response to L1CAM-mediated immunotherapy would be consistent with a crosstalk between L1CAM and the tumor immune microenvironment. Such a hypothesis was further supported in preclinical models of pancreatic cancer. Sebens and collaborators [136] demonstrated that L1CAM induced an immune suppressive phenotype in malignant pancreatic ductal adenocarcinoma (PDAC). In particular, T-regs, but not T-effs, were more prone in migrating on L1CAM expressing H6c7 and Panc1 cells. Moreover, in such microenvironment T-effs reduced their proliferation and inhibited autologous T cell proliferation. L1CAM promoted the establishment of a microenvironment that could favor immune escape and might contribute to tumor progression and chemoresistance [136].

The therapeutic effects of anti-L1CAM antibodies were also assessed in melanoma and pancreatic carcinoma. Doberstein et al. evaluated the therapeutic efficacy of anti-L1CAM treatment on the syngeneic tumor models RET (melanoma) and Panc02 (pancreatic adenocarcinoma), both genetically manipulated to express L1CAM [123]. They also employed the clone L1-9.3 and confirmed both the antibody-mediated reduction of tumor growth and the involvement of immune effector mechanisms in its anti-tumor effect. In addition, the authors showed that anti-L1CAM treatment induced EMT via EGFR phosphorylation. Another study reported cytotoxicity and anti-tumor activity of L1CAM chimeric antibody cA10-A3 in a mouse model of intrahepatic cholangiocarcinoma [124]. Of note, despite L1CAM expression in the nervous system and other body districts, no signs of behavioral changes or other signs of toxicity were reported with the use of anti-L1CAM antibodies for all the studies presented here. This supports the potential clinical utility of such tools as antitumor strategy.

As outlined in Section 4.1, L1CAM is causally related to chemoresistance in various cancer types, which suggests that interfering with L1CAM function may enhance the response to other treatments. Indeed, anti-L1CAM antibodies improved the efficacy of chemotherapeutic drugs in preclinical models of ovarian and pancreatic cancer [137]. The authors employed two different anti-L1CAM antibodies: L1-14.10 and L1-9.3. Pancreatic adenocarcinoma Colo357 and ovarian cancer SKOV3ip cell lines were treated with both antibodies in combination with either gemcitabine or paclitaxel, respectively. The addition of anti-L1CAM inhibited tumor growth much more than chemotherapy alone and increased apoptosis. Lower levels of NF-κB were observed upon combo treatment along with a reduction in vascular endothelial growth factor (VEGF) production and CD31-positive vessels. To note, increased monocyte infiltration was also observed in this study upon combination treatment. Along the same line, Cho et al. have demonstrated that treating intrahepatic cholangiocarcinoma (Choi-CK) xenograft mouse model with gemcitabine or cisplatin in combination with anti-L1CAM antibody Ab417 inhibited tumor growth [126]. The combination of L1CAM antibodies with conventional chemotherapy or other targeted approaches remains an area of research that may have relevant implications and deserves further investigation.

### 5.2. Radioimmunoconjugates

Besides these strategies for functional inactivation, the expression pattern of L1CAM in cancer suggests that anti-L1CAM antibodies may also be harnessed to deliver cytotoxic agents to tumor cells. It is rather surprising, in this regard, that no reports are available in the literature about antibody-drug conjugates based on L1CAM. In fact, L1CAM undergoes endocytosis [39], which is enhanced by antibody binding [138,139], supporting the hypothesis that antibody-drug conjugates would represent suitable tools against L1CAM-expressing cancer cells.

Anti-L1CAM antibodies, instead, have been widely used in preclinical models of ovarian carcinoma and other tumor types as radioimmunotherapy tools upon conjugation with different radioactive isotopes (Figure 4). Fischer et al. used a chimeric anti-L1CAM monoclonal antibody (chCE7) conjugated with 1,4,7,10-tetraazacyclododecane-N-N’-N’-N‴-tetra acetic acid (DOTA) and labeled with the low-energy β-emitter lutetium-177 (^177^Lu) to treat human ovarian cancer-bearing mice. A single treatment with ^177^Lu-DOTA-chCE7 was able to increase mice survival upon subcutaneous injection of SKOV3.ip1 [127]. Grünberg and coworkers showed that the treatment of nude mice bearing IGROV1 xenografts with terbium-161-labelled chCE7 increased radiotoxicity in respect to the radioimmunoconjugate alone [128]. In the neuroblastoma model SK-N-SH, the efficacy of Iodine-131 (^131^I)-labelled chCE7 was compared with that of ^131^I-metaiodobenzylguanidine (MIBG), currently used for treating recurrent or refractory neuroblastoma [129]. ^131^I-MIBG was less effective than ^131^I-chCE7 in reducing tumor volume although none of them abolished tumor growth. Both treatments elicited a transient response since, after the initial reduction, more pronounced for ^131^I-chCE7, the tumor started to regrow reaching the starting volume. Interestingly, both molecules were also tested as imaging tools in seven patients with recurrent neuroblastoma. The two imaging approaches were complementary in targeting the tumor and therefore the authors proposed their use in a combined radioimmunotherapy [129]. Another group has recently conjugated the anti-L1CAM antibody cA10-A3 with 1,4,7-triazacyclononane-1,4,7-triacetic acid (NOTA) labelled with ^177^Lu and evaluated its efficacy in cholangiocarcinoma [130]. The treatment with ^177^Lu-NOTA-cA10-A3 of mice xenografted with L1CAM-overexpressing SCK-L1 cells reduced tumor volume by promoting cell apoptosis and reducing cell proliferation.

In addition, radio-labelled L1CAM antibodies have also been employed together with other drugs. Lindenblatt et al. coupled ^177^Lu-DOTA-chCE7 with paclitaxel treatment. They reported a synergistic inhibitory effect on IGROV1 viability via cell cycle arrest in the radiosensitive G2/M phase [131]. The combined treatment prolonged survival and also delayed tumor latency in vivo. In another study, the same group combined ^177^Lu-DOTA-chCE7 with protein kinase inhibitors (PKIs) to treat ovarian cancer [132]. Among five compounds (alisertib, MK1775, MK2206, saracatinib, temsirolimus), they selected MK1775 for further characterization due to its higher efficacy. The combination of MK1775 with ^177^Lu-DOTA-chCE7, administering either the PKI firstly or both compounds at the same time, synergistically reduced IGROV1 proliferation. The treatment induced DNA double strand breaks in IGROV1 cells, as reflected by histone H2A.X phosphorylation at Ser-139 (γH2A.X), resulting in tumor cell apoptosis. γH2A.X foci were also found in SKOV3ip xenografts upon treatment with either MK1775 alone or in combination with the radioimmunoconjugate. The combined treatment reduced tumor volume in mouse xenografts with respect to the PKI alone, even if the treatment with only ^177^Lu-DOTA-chCE7 was sufficient by itself to reduce tumor growth to a similar extent [132]. A comparison of these findings with those obtained by combining ^177^Lu-DOTA-chCE7 with paclitaxel (see above) will help to inform future therapeutic strategies. Overall, these data support the feasibility of combination treatments based on L1CAM radioimmunoconjugates as efficacious strategies.

Besides radioimmunotherapy, the conjugation of L1CAM antibodies with various radioisotopes has generated reagents that found interesting applications in tumor imaging. Indeed, the successful detection of neoplastic lesions has been reported not only in preclinical cancer models [130,140,141] but also in patients as described above [129]. Due to the expression and function of L1CAM in cancer stem cells for some tumors, the possibility of imaging this cell subpopulation might be of help not only for prognostic purposes but also to monitor the evolution of the disease.

### 5.3. CAR-T Cells

L1CAM has also been investigated as a target for cellular therapies based on the adoptive transfer of chimeric antigen receptor-redirected T (CAR-T) cells (Figure 4). This approach dates back to 1993 and was first published by the immunologist Zelig Eshhar [142]. Since its first application, CAR-T cell therapy proved to be an intriguing approach and today has become of paramount importance for liquid cancers where it offers a successful therapeutic strategy [143,144]. It is based on the ex vivo isolation of tumor-reactive T lymphocytes engineered to express a chimeric antigen to redirect against the tumor. However, such a strategy quickly became of interest for its potential also in solid tumors where it was pioneered by Jensen et al. in neuroblastoma patients [133]. The authors generated CAR-T cells co-expressing an L1CAM-specific chimeric receptor (CE7R) and the fusion gene encoding the selection-suicide enzyme hygromycin phosphotransferase–thymidine kinase (HyTK). These CAR-T cells were then tested in a phase-I clinical trial for children with recurrent/refractory neuroblastoma. Treated patients did not show any sign of overt toxicity. Despite that the study’s primary endpoint was limited to safety of the treatment, the authors also monitored patients’ response. Among the six patients who underwent adoptive CAR-T cell therapy, one patient had first a stable disease and then a partial response, one patient displayed a complete response and another patient had stable disease. However, among the six patients only one experienced a prolonged survival. Subsequent studies also supported CAR-T therapy feasibility in other neoplasms. Hong et al. evaluated the applicability of L1CAM-based CAR-T cells in ovarian cancer [134]. The authors showed that CE7R T-cells were able to kill a panel of human ovarian cancer cell lines. Moreover, CE7R T-cells also recognized and killed ascites-derived primary cells. In SKOV3 xenograft mice, the treatment significantly inhibited tumor growth and reduced ascites production compared to control mice. Andersch et al. employed CAR-T cells co-targeting both L1CAM and the GD2 ganglioside in retinoblastoma cell lines, and reported that this approach led to elimination of tumor cells in vitro [135]. Interestingly, they also described an escape mechanism whereby retinoblastoma cells downregulate the expression of both antigens after CAR-T treatment. Further validation of this approach using in vivo preclinical models will undoubtedly provide important insights into its clinical utility.

In summary, various studies conducted on different tumor types have shown the remarkable potential of L1CAM-targeting strategies for the design of innovative antitumor therapies.

## 6. Potential Implications and Clinical Perspectives of L1CAM in Cancer and CSC

Many lines of experimental and clinical evidence support the potential relevance of L1CAM in the management of tumor patients. For example, as outlined in Section 2.2.2, the ectodomain of L1CAM is released into the extracellular space upon proteolytic cleavage. This raises the possibility that shed L1CAM becomes detectable in blood and other body fluids. Indeed, several studies found a correlation between soluble L1CAM levels in liquid biopsies from cancer patients and different clinical parameters [57,61,144,145]. Along these lines, we have recently found a novel soluble isoform of L1CAM, endowed with angiogenic functions, that lacks the transmembrane domain and is released in the extracellular space. The levels of this isoform resulted in being associated with vessel density in ovarian cancer specimens suggesting an implication also in tumor angiogenesis [52]. Thus, L1CAM is a promising non-invasive biomarker with diagnostic and prognostic value.

The expression pattern of L1CAM in many different cancer types as well as its pivotal role in diverse tumor-related cellular processes, including cancer stemness, makes it a suitable target for antitumor therapies. Indeed, L1CAM-targeted therapies resulted in multiple advantages, among which are counteracting tumor cell invasion, EMT and metastasis initiation, and pro-malignant interactions between tumor cells and their microenvironment. Based on the studies discussed in the previous sections, this list should also include the inhibition of CSC function.

Yet, with the exception of a phase-I trial with CAR-T cells in neuroblastoma patients ([133], see also Section 5), to date no L1CAM-based treatments have reached clinical use, although there are biotech companies that are actively pursuing this goal. Future efforts in this direction should take into account various aspects, including, for example, the potential side effects of L1CAM-targeted treatments. The protein, indeed, is abundantly expressed in the nervous system as well as in other cell types of various organs (e.g., the hematopoietic system and various epithelia). While no studies on preclinical models have reported signs of overt toxicity of anti-L1CAM agents, additional early-phase clinical trials are warranted to validate these findings in humans.

Finally, by analogy to several targeted treatments, it is unlikely that any L1CAM-based strategy would give satisfactory results as a monotherapy. We believe that L1CAM-targeted treatments should be considered in the context of combination therapies. This view is supported, for example, by the reported role of L1CAM in conferring chemoresistance to cancer cells (see Section 4.2), which implies that neutralizing L1CAM would restore sensitivity to conventional antitumor drugs. Furthermore, emerging evidence (mentioned in Section 5.1) also points to a crosstalk of L1CAM with the tumor immune microenvironment which contributes to immune evasion. Hence, one can speculate that L1CAM inactivation synergizes with immunotherapy in overcoming the ability of many tumors to escape the immune attack.

So far, L1CAM has not been investigated as a target in the context of therapies directed against CSC. Yet the expression pattern and the role of the molecule in this subpopulation of tumor cells provide the rationale for assessing the impact of L1CAM-targeted treatments on CSC function. Indeed, L1CAM may represent an Achilles heel for such a cell population. As outlined above, a few strategies have been designed to either interfere with L1CAM activity or deliver antitumor agents to L1CAM-expressing cells. Thus, it will be of interest to test whether such strategies impact on tumor stemness and on CSC-related properties such as self-renewal, chemoresistance and cancer initiation. Given the role of L1CAM in CSC, it is intriguing to envisage L1CAM-based strategies to target this cancer cell subpopulation. However, based on the functional contribution of CSC to tumor metastasis and relapse, the therapeutic window becomes a key factor in order to achieve better efficacy. One can expect, in this regard, a lower effect in the neoadjuvant treatment of a primary tumor as compared to the prevention of metastatic dissemination or tumor recurrence.

Future research will clarify if and to what extent L1CAM targeting represents a suitable strategy for innovative antitumor therapies, focusing in particular on the opportunity of defeating CSC-driven metastasis, relapse and drug resistance, thus contributing to tumor eradication.

## Figures and Tables

**Table 1:** Homophilic and heterophilic L1CAM interactors. ND = not defined. *not conclusively demonstrated but inferred from the data provided in the corresponding reference.

Interactors	L1CAM Motif Involved	Type of Interaction	References
L1CAM	Ig1-6, FN2-3	*cis, trans*	[11,15,16,17]
NCAM	Ig5	*cis*	[18,19]
Neurocan	Ig1	ND	[20]
Integrins	Ig6, FN3	*cis*	[17,21,22]
Laminin	Ig6*	*trans*	[23]
Neuropilin	Ig1	*cis, trans*	[10,24]
Ankyrin	Cytoplasmic domain		[25,26]
FGFR	FN3, Ig1, Ig2	*cis, trans*	[27]
EGFR	FN5, Ig3*	*cis, trans*	[28]

**Table 2:** L1CAM clinical relevance in cancer.

Cancer Type	Prognostic Value	Clinico-Pathological Parameters Correlating with L1CAM	References
Endometrial cancer	Negative (OS, DFS)	High grade, lymph node metastasis, tumor relapse	[55,56,57,58,59,60]
Ovarian cancer	Negative (OS, DFS)	Low tumor resectability, lymph node metastasis, chemoresistance,	[61,62,63,64]
Melanoma	Negative (DFS)	Metastasis	[65]
Breast cancer	Negative (DFS)	Larger tumor size, lymph node involvement, higher histologic grade, advanced TNM stage	[66]
Gastric cancer	Negative (OS, DFS)	Distant metastasis	[67]
Colon cancer	Negative (OS)	Advanced cancer stage, distant metastasis and tumor recurrence	[68]
Pancreatic cancer	Negative (OS)	Lymph node involvement, vascular invasion, perineural invasion and higher degree of pain	[69]
Non-small cell lung cancer	Negative (PFS)	None	[70]
Kidney cancer	Negative (OS)	ND	[71]

**Table 3:** L1CAM-targeted therapeutic approaches in different cancers.

Cancer Type	Therapeutic Strategy	Antibody Clone	Effect	References
Ovarian cancer	Antibody alone	chCE7, L1-11A	↓ Proliferation ↓ Migration	[121]
Ovarian cancer	Antibody alone	CE7	↓ Proliferation	[62]
Ovarian cancer	Antibody alone	L1-9.3	↓ Tumor growth ↑ Survival	[122]
Melanoma Pancreatic cancer	Antibody alone	L1-9.3	↓ Tumor growth EMT induction	[123]
Cholangiocarcinoma	Antibody alone	cA10-A3	↓ Tumor growth	[124]
Pancreatic cancer	Antibody + gemcitabine	L1-14.10, L1-9.3	↓ Tumor growth ↑ Apoptosis	[125]
Ovarian cancer	Antibody + paclitaxel	L1-14.10, L1-9.3	↓ Tumor growth ↑ Apoptosis	[125]
Cholangiocarcinoma	Antibody + gemcitabine	Ab417	↓ Tumor growth	[126]
Cholangiocarcinoma	Antibody + cisplatin	Ab417	↓ Tumor growth	[126]
Ovarian cancer	Radioimmunoconjugate	^177^Lu-DOTA-chCE7	↑ Survival	[127]
Ovarian cancer	Radioimmunoconjugate	^161^Tb-chCE7	↓ Tumor growth	[128]
Neuroblastoma	Radioimmunoconjugate	^131^I-chCE7	↓ Tumor growth	[129]
Cholangiocarcinoma	Radioimmunoconjugate	^177^Lu-NOTA-cA10-A3	↓ Tumor growth ↑ Apoptosis ↓ Proliferation	[130]
Ovarian cancer	Radioimmunoconjugate + paclitaxel	^177^Lu-DOTA-chCE7	↑ Survival Tumor growth delay	[131]
Ovarian cancer	Radioimmunoconjugate + protein kinase inhibitor	^177^Lu-DOTA-chCE7	↓ Proliferation ↓ Tumor growth	[132]
Neuroblastoma	CAR-T cell	CE7R	Various responses	[133]
Ovarian cancer	CAR-T cell	CE7R	↓ Tumor growth ↓ Ascites	[134]
Retinoblastoma	CAR-T cell	CE7R	Cytotoxicity	[135]
